# Vitamin D Status of Mice Deficient in Scavenger Receptor Class B Type 1, Cluster Determinant 36 and ATP-Binding Cassette Proteins G5/G8

**DOI:** 10.3390/nu12082169

**Published:** 2020-07-22

**Authors:** Mikis Kiourtzidis, Julia Kühn, Corinna Brandsch, Gabriele I. Stangl

**Affiliations:** 1 Institute of Agricultural and Nutritional Sciences, Martin Luther University Halle-Wittenberg, Von-Danckelmann-Platz 2, 06120 Halle (Saale), Germany; mikis.kiourtzidis@landw.uni-halle.de (M.K.); corinna.brandsch@landw.uni-halle.de (C.B.); gabriele.stangl@landw.uni-halle.de (G.I.S.); 2 Competence Cluster of Cardiovascular Health and Nutrition (nutriCARD), Halle-Jena-Leipzig, Deutscher Pl. 5E, 04103 Leipzig, Germany

**Keywords:** ATP-binding cassette transporters G5/G8, cluster determinant 36, scavenger receptor class B type 1, vitamin D, mice

## Abstract

Classical lipid transporters are suggested to modulate cellular vitamin D uptake. This study investigated the vitamin D levels in serum and tissues of mice deficient in SR-B1 (Srb1^-/-^), CD36 (Cd36^-/-^) and ABC-G5/G8 (Abcg5/g8^-/-^) and compared them with corresponding wild-type (WT) mice. All mice received triple-deuterated vitamin D_3_ (vitamin D_3_-d_3_) for six weeks. All knockout mice vs. WT mice showed specific alterations in their vitamin D concentrations. Srb1^-/-^ mice had higher levels of vitamin D_3_-d_3_ in the serum, adipose tissue, kidney and heart, whereas liver levels of vitamin D_3_-d_3_ remained unaffected. Additionally, Srb1^-/-^ mice had lower levels of deuterated 25-hydroxyvitamin D_3_ (25(OH)D_3_-d_3_) in the serum, liver and kidney compared to WT mice. In contrast, Cd36^-/-^ and WT mice did not differ in the serum and tissue levels of vitamin D_3_-d_3_, but Cd36^-/-^ vs. WT mice were characterized by lower levels of 25(OH)D_3_-d_3_ in the serum, liver and kidney. Finally, Abcg5/g8^-/-^ mice tended to have higher levels of vitamin D_3_-d_3_ in the serum and liver. Major alterations in Abcg5/g8^-/-^ mice were notably higher levels of 25(OH)D_3_-d_3_ in the serum and kidney, accompanied by a higher hepatic mRNA abundance of *Cyp27a1* hydroxylase. To conclude, the current data emphasize the significant role of lipid transporters in the uptake, tissue distribution and activation of vitamin D.

## 1. Introduction

Most vitamin D in the body is synthesized from cutaneous 7-dehydrocholesterol (7-DHC) via ultraviolet B (UVB) light exposure. Due to seasonal fluctuations in the intensity of UVB radiation or situations that limit the endogenous synthesis of vitamin D, many individuals are at high risk of developing vitamin D deficiency. Thus, several health authorities have established guidelines on vitamin D intake to prevent or treat vitamin D deficiency [1,2,3,4,5]. The regular intake of a vitamin D supplement is a common and efficient strategy to improve vitamin D status. However, despite the widespread use of vitamin D supplements, there is insufficient knowledge on the intestinal absorption and cellular uptake of orally ingested vitamin D. In vitro and ex vivo data from Reboul and coworkers indicate a role of cholesterol transporters in vitamin D uptake [6].

Recently, we showed that the sterol transporter Niemann-Pick C1-like protein 1 (NPC1L1) plays a crucial role in vitamin D uptake and storage because mice treated with the NPC1L1 inhibitor ezetimibe showed significantly reduced concentrations of vitamin D in the liver, adipose tissue, the kidney and the heart [7]. However, the contribution of other lipid transporters, such as scavenger receptor class B type 1 (SR-B1), cluster determinant 36 (CD36) and ATP-binding cassette transporters G5 and G8 (ABC-G5/G8), for the uptake of ingested vitamin D are still uncertain.

A putative membrane receptor for vitamin D uptake is SR-B1. It normally functions as a high-density lipoprotein (HDL) receptor and is pivotal for the uptake of cholesterol from peripheral tissues back into the liver. However, SR-B1 is expressed not only in the liver but also in many different types of cells, such as enterocytes, macrophages, endothelial cells, and adipocytes (reviewed by Shen et al. [8]). Previous data show that SR-B1 is involved in the uptake of tocopherols and carotenoids [9,10,11]. Reboul et al. further demonstrated that mice overexpressing intestinal SR-B1 had markedly higher intestinal uptake of vitamin D than wild-type (WT) mice [6]. The role of SR-B1 in tissue storage and the status of vitamin D is currently not known.

A second transporter, which might be important for the uptake and tissue distribution of vitamin D, is CD36. CD36 has a significant role in lipid homeostasis, as it is involved in fatty acid uptake in enterocytes [12], cardiomyocytes [13,14], and adipocytes [14] but not in hepatocytes [14]. In 2011, Reboul et al. demonstrated that transfection of human embryonic kidney (HEK) cells with human CD36 significantly increased the uptake of vitamin D [6].

Finally, the ABC transporter G family could be relevant in vitamin D metabolism. ABC-G5 and ABC-G8 form a heterodimeric complex located in the brush-border membrane of enterocytes, the canalicular membranes of hepatocytes and the membranes of epithelial cells in the gallbladder [15]. They are responsible for the efflux of nonesterified sterols, such as plant sterols and, to a minor extent, cholesterol, from enterocytes back into the intestinal lumen (for review, see Zein et al. [16]). In the liver, ABC-G5/G8 facilitates the excretion of sterols into bile [17]. Mutations in either of these two genes causes sitosterolemia, which is characterized by increased intestinal absorption and decreased biliary secretion of plant sterols [18]. On the other hand, plant sterols can stimulate the intestinal expression of ABC-G5/G8 [19]. A possible role of ABC-G5/G8 in vitamin D absorption was described by Goncalves et al., who showed that phytosterols can impair vitamin D intestinal absorption in vitro and in mice [20]. In contrast, a meta-analysis of human trials performed in 2003 showed no changes in circulating vitamin D after the consumption of plant sterols and stanol esters [21]. Thus, the significance of ABC-G5/G8 in vitamin D metabolism remains obscure.

Because data concerning the roles of SR-B1, CD36 and ABC subtype G transporters for vitamin D uptake, tissue distribution and activation are scarce, we conducted studies with mice that were deficient in SR-B1, CD36 or ABC-G5/G8. This is also relevant as polymorphisms of these transporters and their impact on lipid metabolism have been described in humans [22,23,24]. To elucidate the fate of orally administered vitamin D in these mouse models, we used standardized diets with triple-deuterated vitamin D_3_ (vitamin D_3_-d_3_).

## 2. Materials and Methods

### 2.1. Animals and Feeding

The experimental design of this study was planned and performed in consideration of animal welfare standards according to the US National Research Council (NRC) [25]. It was approved by the animal welfare authority of Saxony-Anhalt (Landesverwaltungsamt Halle (Saale), Germany, approval number: 42502-2-1461 MLU, date of approval: 19 April 2018). Three different knockout mouse models and two corresponding WT mouse models were included in the study. Each group consisted of 5–7 male mice. Sample size was calculated using G*Power software (version 3.1.9.6) to determine statistically significant differences in the tissue levels of vitamin D_3_-d_3_. Recently published data on the importance of the NPC1L1 transporter for the uptake and tissue distribution of vitamin D in mice treated with ezetimibe [7] were used as a basis for sample size calculation. Using an estimated effect size of 3,76 (Cohen’s d), a significance level of 5% and a power of 0.95, a minimum of 5 mice per group was considered to be a sufficient sample size. Homozygous Srb1 knockout (Srb1^-/-^) mice were obtained by mating mice heterozygous for Srb1 generated by Rigotti et al. [26] (Srb1^+/-^, B6;129S2-*Scarb1^tm1Kri^*/J, #003379, The Jackson Laboratory, Bar Harbor, ME, USA). Cd36 knockout (Cd36^-/-^) mice (B6.129S1-*Cd36^tm1Mfe^*/J) were supplied by the Jackson Laboratory (#019006). Male WT C57BL/6J mice served as the appropriate control for Srb1^-/-^ and Cd36^-/-^ mice and were also purchased from the Jackson Laboratory (#000664). Abcg5/g8 double knockout (Abcg5/g8^-/-^) mice (B6;129S6-Del(17Abcg5-Abcg8)1Hobb/J) were bred in our facility by mating homozygous male Abcg5/g8^-/-^ mice with heterozygous females (Abcg5/g8^+/-^). The breeding pairs were obtained from the Jackson Laboratory (#004670). Homozygous Abcg5/g8^+/+^ mice were used as the corresponding WT control.

All mice were housed individually in Macrolon cages in a climatic room (22 ± 2 °C, 50–60% humidity), with a 12/12 h light-dark cycle. To avoid the endogenous synthesis of vitamin D, we used lamps that did not emit UV light.

Before starting the study, all mice received a diet with 25 µg (1000 IU) of vitamin D_3_/kg for one week to standardize their vitamin D status. Subsequently, mice aged 6 to 8 weeks were fed a standardized diet containing 25 µg/kg (1000 IU/kg) vitamin D_3_-d_3_ (Sigma-Aldrich, Steinheim, Germany) as the only source of vitamin D for six weeks. This feeding period has been shown to be long enough to induce distinct changes in vitamin D metabolism [7]. The use of deuterated vitamin D ensures a closer tracking of ingested vitamin D and its metabolic route in the body. To ensure that all mice are supplied with the same amount of vitamin D_3_-d_3_, the diet was restricted to 2.4 g per day. The supplied amount of diet corresponds to 80% of the *ad libitum* food intake. Since Srb1^-/-^ mice did not fully ingest their provided food, their mean food intake was 2.2 ± 0.2 g/d. The concentration of vitamin D_3_-d_3_ in the diet met the recommendations for vitamin D in growing mice [27]. The experimental diet (per kg) consisted of 288 g starch, 200 g sucrose, 200 g casein, 175 g coconut fat, 25 g soybean oil, 50 g cellulose, 60 g of a mineral-vitamin-mixture that contains, except vitamin D, quantities of vitamins and minerals according to the NRC [27]) and 2 g DL-methionine. All mice had free access to water.

### 2.2. Sample Collection

After six weeks of feeding the vitamin D_3_-d_3_-containing diets, mice were anaesthetized and decapitated after 4 h of fasting. Blood was collected in serum tubes (Sarstedt, Nümbrecht, Germany) for analyses of cholesterol, triglycerides, transaminases and vitamin D metabolites. As we recently demonstrated that liver, retroperitoneal adipose tissue, kidney and heart are the major tissues showing substantial quantities of vitamin D [7], we decided to use these tissues to analyze the concentrations of deuterium-labeled vitamin D_3_ metabolites. The liver was additionally used for analyses of cholesterol and triglycerides. The mRNA abundance of genes involved in vitamin D hydroxylation was analyzed in the liver and kidney. After snap freezing the tissues in liquid nitrogen, they were stored together with the serum samples at −80 °C until analysis.

### 2.3. Analysis of Cholesterol and Triglycerides

Cholesterol and triglycerides were quantified in serum and liver samples with the Diagnostic Systems assay (Holzheim, Germany). Preparation of the liver samples was done as described elsewhere [28].

### 2.4. Analysis of Transaminases

As biomarkers of hepatic injury, aspartate aminotransferase (ASAT) and alanine aminotransferase (ALAT) were measured in serum using a commercial photometric assay (Diagnostic Systems).

### 2.5. Analysis of 7-Dehydrocholesterol and Vitamin D Metabolites

The concentrations of 7-DHC, vitamin D_3_-d_3_, and triple-deuterated 25-hydroxyvitamin D_3_ (25(OH)D_3_-d_3_) were quantified in serum and tissues by liquid chromatography–tandem mass spectrometry (LC–MS/MS). Sample preparation and quantification of the sterols were performed as described recently [7]. Mass transitions were (as adducts of 4-phenyl-1,2,4-triazoline-3,5-dione; Sigma-Aldrich): 7-DHC 560 > 365, 7-fold deuterated 7-DHC 567 > 372, vitamin D_3_-d_3_ 563 > 301, 7-fold deuterated vitamin D_3_ 567 > 298, 25(OH)D_3_-d_3_ 579 > 301, and 6-fold deuterated 25(OH)D_3_ 582 > 298. The limit of quantification, which was defined as a signal-to-noise ratio above 10, for 25(OH)D_3_-d_3_ was 3.0 pmol/g in adipose tissue and 3.2 pmol/g in the heart. Concentrations of the other vitamin D metabolites in serum or tissues were present in reliably measurable ranges.

The serum concentration of 1α,25-dihydroxyvitamin D (1,25(OH)_2_D) was analyzed by a commercial ELISA (Immunodiagnostic Systems, Frankfurt am Main, Germany). The protocol was given by the manufacturer.

### 2.6. Analysis of Relative mRNA Abundance

The relative mRNA abundance of genes involved in vitamin D hydroxylation was analyzed in the liver and kidney using real-time RT-PCR. The complete procedure, including trizol-based extraction of total RNA, cDNA synthesis and a detailed protocol of the RT-PCR was previously described [29]. The modified ∆∆CT method of Pfaffl was used for the calculation of the relative mRNA concentration [30]. For normalization, glyceraldehyde-3-phosphate dehydrogenase (*Gapdh*, XM_001473623, purchased from Eurofins Genomics, Ebersberg, Germany), ribosomal protein lateral stalk subunit P0 (*Rplp0*, NM_007475, Eurofins Genomics) and hypoxanthine phosphoribosyltransferase (*Hprt*, NM_013556.2, Sigma-Aldrich) were used as reference genes. Primer pairs for sterol 27-hydroxylase (*Cyp27a1*, NM_024264), vitamin D 25-hydroxylase (*Cyp2r1*, NM_177382) and 25-hydroxyvitamin D 1α-hydroxylase (*Cyp27b1*, NM_010009.2) were purchased from Sigma-Aldrich (www.kicqstart-primers-sigmaaldrich.com).

### 2.7. Statistical Analysis

All data are presented as the means ± standard deviations (SD). The SPSS version 25.0 (IBM, Armonk, NY, USA) was used for statistical data treatment. Because data were normally distributed (Shapiro–Wilk test), each group of knockout mice was compared with the corresponding group of WT mice by using Student’s *t*-test. *p* < 0.05 was considered significantly different.

## 3. Results

### 3.1. Body Weight, Lipids and Serum Transaminases

The final body weights and weight gain of Cd36^-/-^ mice and Abcg5/g8^-/-^ mice did not differ from those of their corresponding WT mice; however, the final body weights of Srb1^-/-^ mice were slightly lower than those of the corresponding WT mice, which was attributed to the moderately lower food intake in this group (*p* < 0.05; Table 1). No differences in the absolute and relative liver weights were observed between the Srb1^-/-^ or Cd36^-/-^ mice and their corresponding WT counterparts, whereas higher absolute and relative liver weights were observed in Abcg5/g8^-/-^ mice than in the corresponding WT mice (Table 1). Serum levels of ASAT and ALAT did not differ between knockout mice and the corresponding WT mice (Table 1), assuming that the higher liver weights of Abcg5/g8^-/-^ mice are not indicative of liver injury.

To elucidate the impact of SR-B1, CD36 and ABC-G5/G8 deficiency on lipid status, we analyzed serum concentrations of triglycerides, cholesterol and 7-DHC and liver levels of triglycerides and cholesterol. Srb1^-/-^ mice had significantly higher concentrations of triglycerides (*p* < 0.01), cholesterol (*p* < 0.001) and 7-DHC (*p* < 0.001) in serum than WT mice, whereas the levels of triglycerides and cholesterol in the liver did not differ between the two groups of mice (Table 1).

In contrast, Cd36^-/-^ mice were characterized by lower serum levels of triglycerides (*p* = 0.001) and higher serum levels of cholesterol (*p* < 0.01) and 7-DHC (*p* < 0.01) than WT mice. Compared to WT mice, liver levels of cholesterol were lower in Cd36^-/-^ mice than in WT mice (*p* < 0.05); triglyceride levels in the livers of Cd36^-/-^ mice were 1.5-fold higher than those of the WT mice, although this difference did not reach significance level (Table 1).

Finally, the serum concentrations of triglycerides, cholesterol and 7-DHC in Abcg5/g8^-/-^ mice were distinctly higher than those in the corresponding WT mice (*p* < 0.001, *p* = 0.001, *p* < 0.05), whereas the liver levels of cholesterol tended to be lower in Abcg5/g8^-/-^ mice than in WT mice (*p* = 0.067; Table 1). Liver triglycerides did not differ between these two groups (Table 1).

### 3.2. Concentrations of Deuterium-Labeled Vitamin D_3_ in Serum and Tissues

To investigate the impacts of SR-B1, CD36 and ABC-G5/G8 on vitamin D tissue distribution and serum vitamin D levels, we first quantified the concentrations of deuterium-labeled vitamin D_3_ in the serum, liver, retroperitoneal adipose tissue, kidney and heart of Srb1^-/-^, Cd36^-/-^ and Abcg5/g8^-/-^ mice and compared them with those of the corresponding WT mice.

**Figure 1:**
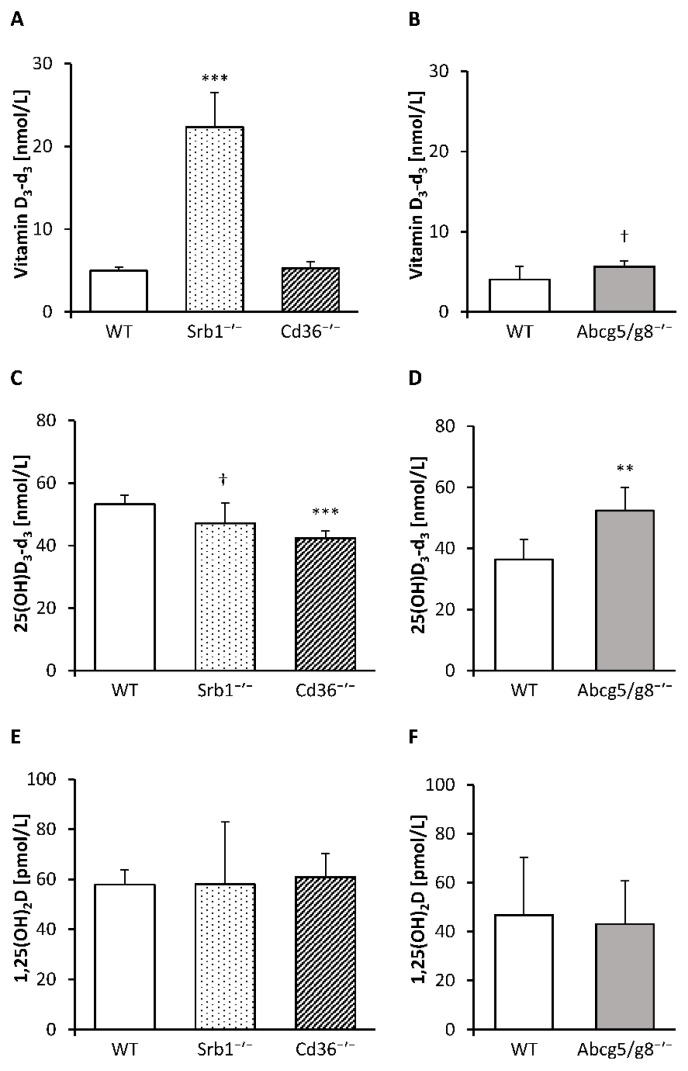
Serum concentrations of vitamin D_3_-d_3_ in Srb1^-/-^ and Cd36^-/-^ mice versus corresponding wild-type (WT) mice (**A**) and Abcg5/g8^-/-^ mice versus corresponding WT mice (**B**). Serum concentrations of 25(OH)D_3_-d_3_ in Srb1^-/-^ and Cd36^-/-^ mice versus corresponding WT mice (**C**) and Abcg5/g8^-/-^ mice versus corresponding WT mice (**D**). Serum concentrations of 1,25(OH)_2_D in Srb1^-/-^ and Cd36^-/-^ mice versus corresponding WT mice (**E**) and Abcg5/g8^-/-^ mice versus corresponding WT mice (**F**). All mice were fed diets containing 25 µg/kg triple-deuterated vitamin D_3_ (vitamin D_3_-d_3_) for six weeks. Data are presented as the means ± SD, *n* = 5–7; *** *p* < 0.001, ** *p* < 0.01, ^†^ *p* < 0.1 (compared to the corresponding WT mice, Student’s *t* test). 1,25(OH)_2_D, 1α,25-dihydroxyvitamin D; 25(OH)D_3_-d_3_, triple-deuterated 25-hydroxyvitamin D_3_; Abcg5/g8^-/-^, ATP-binding cassette transporters G5/G8 knockout mice; Cd36^-/-^, cluster determinant 36 knockout mice; Srb1^-/-^, scavenger receptor class B type 1 knockout mice.

**Figure 2:**
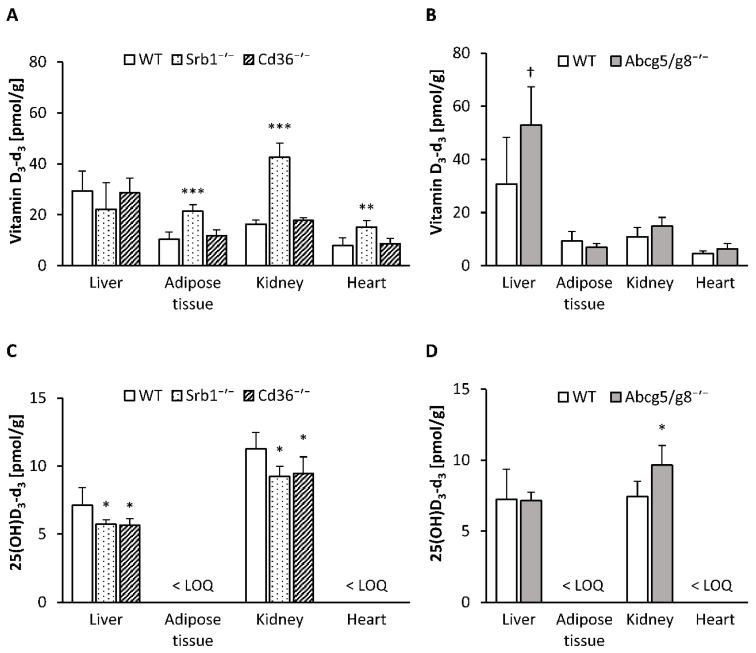
Concentrations of vitamin D_3_-d_3_ in the liver, retroperitoneal adipose tissue, kidney and heart of Srb1^-/-^ and Cd36^-/-^ mice versus corresponding wild-type (WT) mice (**A**), and Abcg5/g8^-/-^ mice versus corresponding WT mice (**B**). Concentrations of 25(OH)D_3_-d_3_ in the liver, retroperitoneal adipose tissue, kidney and heart of Srb1^-/-^ and Cd36^-/-^ mice versus corresponding WT mice (**C**), and Abcg5/g8^-/-^ mice versus corresponding WT mice (**D**). All mice were fed diets containing 25 µg/kg triple-deuterated vitamin D_3_ (vitamin D_3_-d_3_) for six weeks. Data are presented as the means ± SD, *n* = 5–7; *** *p* < 0.001, ** *p* < 0.01, * *p* < 0.05, ^†^ *p* < 0.1 (compared to the corresponding WT mice, Student’s *t* test). The limit of quantification (LOQ) of 25(OH)D_3_-d_3_ was 3.0 pmol/g for the retroperitoneal adipose tissue and 3.2 pmol/g for the heart. 25(OH)D_3_-d_3_, triple-deuterated 25-hydroxyvitamin D_3_; Abcg5/g8^-/-^, ATP-binding cassette transporters G5/G8 knockout mice; Cd36^-/-^, cluster determinant 36 knockout mice; Srb1^-/-^, scavenger receptor class B type 1 knockout mice.

Here, we found markedly higher concentrations of vitamin D_3_-d_3_ in the serum (*p* < 0.001; Figure 1A), retroperitoneal adipose tissue (*p* < 0.001), kidney (*p* < 0.001) and heart (*p* < 0.01) of Srb1^-/-^ mice than in those of WT mice, whereas the liver concentration of vitamin D_3_-d_3_ did not differ between these two groups of mice (Figure 2A). In contrast, no significant differences in the vitamin D_3_-d_3_ concentrations in the serum (Figure 1A), liver, retroperitoneal adipose tissue, kidney or heart (Figure 2A) were observed between the Cd36^-/-^ and WT mice.

Abcg5/g8^-/-^ mice in comparison to WT mice tended to have higher concentrations of vitamin D_3_-d_3_ in the serum (*p* = 0.056; Figure 1B) and liver (*p* = 0.054; Figure 2B), whereas no differences in vitamin D_3_-d_3_ were found in the retroperitoneal adipose tissue, kidney or heart (Figure 2B).

### 3.3. Serum and Tissue Levels of Hydroxylated Vitamin D_3_ Metabolites and mRNA Abundance of Hepatic and Renal Hydroxylases

To elucidate the impacts of SR-B1, CD36 and ABC-G5/G8 deficiency on hydroxylated vitamin D_3_ metabolites, we analyzed 25(OH)D_3_-d_3_ in serum and tissues and the circulating levels of 1,25(OH)_2_D, the bioactive form of vitamin D.

In comparison to WT mice, Srb1^-/-^ mice had slightly lower levels of 25(OH)D_3_-d_3_ in serum (*p* = 0.083; Figure 1C) and significantly lower levels of 25(OH)D_3_-d_3_ in the liver (*p* < 0.05) and kidney (*p* = 0.010; Figure 2C). In Cd36^-/-^ mice, the 25(OH)D_3_-d_3_ concentrations in the serum, liver and kidney were also significantly lower than those in the WT mice (*p* < 0.001, *p* < 0.05, *p* < 0.05, respectively; Figure 1C and Figure 2C). Finally, Abcg5/g8^-/-^ mice, compared to WT mice, were characterized by higher concentrations of 25(OH)D_3_-d_3_ in the serum (*p* < 0.01) and kidney (*p* < 0.05) but not in the liver (Figure 1D and Figure 2D). The serum concentrations of 1,25(OH)_2_D were not differentially influenced by the genotype (Figure 1E,F).

**Figure 3:**
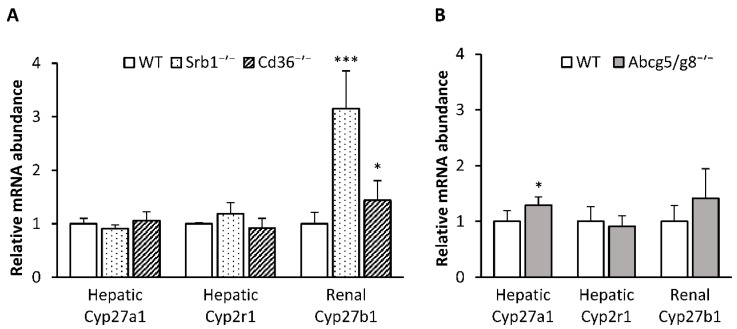
Relative mRNA abundance of hydroxylases in the liver and kidney of Srb1^-/-^ and Cd36^-/-^ mice versus corresponding wild-type (WT) mice (**A**), and Abcg5/g8^-/-^ mice versus corresponding WT mice (**B**). All mice were fed diets containing 25 µg/kg triple-deuterated vitamin D_3_ (vitamin D_3_-d_3_) for six weeks. Data are presented as the means ± SD, *n* = 5–7; *** *p* < 0.001, * *p* < 0.05 (compared to the corresponding WT mice, Student’s *t* test). Abcg5/g8^-/-^, ATP-binding cassette transporters G5/G8 knockout mice; Cd36^-/-^, cluster determinant 36 knockout mice; Cyp27a1, sterol 27-hydroxylase; Cyp27b1, 25-hydroxyvitamin D 1α-hydroxylase; Cyp2r1, vitamin D 25-hydroxylase; Srb1^-/-^, scavenger receptor class B type 1 knockout mice.

To ascertain whether hepatic enzymes involved in the conversion of vitamin D to 25(OH)D were influenced by the genotype, we analyzed the mRNA abundances of *Cyp27a1* and *Cyp2r1*, which are crucial for 25(OH)D synthesis. The data show that the relative mRNA expression levels of both genes in the liver were not significantly affected by SR-B1 and CD36 because Srb1^-/-^ mice and Cd36^-/-^ mice did not show differences in the mRNA abundance of *Cyp27a1* and *Cyp2r1* from that of the WT mice (Figure 3A). In contrast, Abcg5/g8^-/-^ mice were characterized by a moderately higher mRNA abundance of *Cyp27a1* in their livers than the corresponding WT mice (*p* < 0.05), whereas the mRNA abundance of *Cyp2r1* was not different from that of WT mice (Figure 3B). Besides hepatic hydroxylases, we analyzed the mRNA abundance of the renal *Cyp27b1*, the key enzyme for the synthesis of 1,25(OH)_2_D. Analysis revealed that both Srb1^-/-^ mice (*p* < 0.001) and Cd36^-/-^ mice (*p* < 0.05) had significantly higher *Cyp27b1* mRNA abundance in their kidneys than the corresponding WT controls (Figure 3A). In contrast, mRNA abundance of renal *Cyp27b1* was not affected in Abcg5/g8^-/-^ mice (Figure 3B).

## 4. Discussion

The current study aimed to investigate the levels of vitamin D metabolites in tissues and serum of mice deficient in SR-B1, CD36 and ABC-G5/G8. These proteins are primarily involved in the transport of lipids. However, some data, mainly from in vitro studies, have indicated that these transporters could also play an important role in the uptake and tissue distribution of fat-soluble vitamins, including vitamin D. To our knowledge, this is the first study that investigated the impact of these transporters in mice with global SR-B1, CD36 or ABC-G5/G8 knockouts. To track the metabolic route of vitamin D given orally, we used diets with deuterium-labeled vitamin D and controlled the amount of ingested food to avoid pronounced differences in the intake of this vitamin.

The first transporter that we focused on was SR-B1. The high serum levels of lipids, in particular cholesterol, in Srb1^-/-^ mice vs. WT mice fits well with the commonly known function of SR-B1 as an HDL receptor that primarily mediates the uptake of HDL lipids, in particular cholesterol, from circulation into the liver [26,31,32]. The impaired uptake of peripheral cholesterol into liver became further evident by the extremely high serum concentration of 7-DHC in the Srb1^-/-^ mice, which indicates a stimulated cholesterol synthesis as a regulatory measure to counteract a decline in cellular cholesterol levels [33,34]. Main alterations in the vitamin D metabolism of Srb1^-/-^ mice in comparison to the WT mice were a pronounced accumulation of vitamin D_3_-d_3_ (approximately fivefold) and a slight reduction of 25(OH)D_3_-d_3_ in serum. These findings indicate that SR-B1 is not only crucial for the hepatic uptake of HDL cholesterol but is also very important for the uptake of vitamin D from circulation into the liver to synthesize 25(OH)D, the primary biomarker of vitamin D status. We further assume a significant role of HDL for vitamin D transport, because the observed accumulation of serum vitamin D_3_-d_3_ in the Srb1^-/-^ mice paralleled the accumulation of circulating cholesterol. The high vitamin D_3_-d_3_ levels in tissues other than the liver in the Srb1^-/-^ mice can be attributed to the high availability of vitamin D_3_-d_3_ in circulation or the impaired reverse transport of peripheral vitamin D into the liver. It is important to note that the diminished uptake of vitamin D in the livers of Srb1^-/-^ mice was not associated with an increase in the mRNA expression of the hepatic vitamin D hydroxylases *Cyp27a1* and *Cyp2r1*. Data indicate that the reduced availability of 25(OH)D_3_-d_3_ in the Srb1^-/-^ mice had caused an increase in the mRNA expression of the renal *Cyp27b1* to prevent a decline in bioactive 1,25(OH)_2_D. To sum up, the current data indicate an important function of SR-B1 for the uptake of circulating vitamin D into liver. However, the findings were not indicative of a significant importance of SR-B1 for intestinal vitamin D absorption, which has previously been observed in the proximal intestinal fragments of mice overexpressing SR-B1 [6].

The second transporter that we analyzed was CD36. The role of CD36 in lipid metabolism is currently a subject of controversial discussion. It is suggested that CD36 has multiple functions in the lipid metabolism. Thus, the overall importance of CD36 for lipid metabolism can be best investigated by use of a global CD36 knockout model, CD36 is assumed to facilitate the cellular uptake of fatty acids [35,36], which may explain the high expression of CD36 in tissues with pronounced fatty acid metabolism, such as adipose tissue and the heart [37]. It appears to be also involved in hepatic cholesterol uptake [38] and biliary cholesterol transport [39]. The increased serum cholesterol and reduced levels of liver cholesterol which we observed in the Cd36^-/-^ mice are consistent with data obtained from Cd36^-/-^ mice [38] and emphasize the impaired hepatic sterol metabolism in these mice. Other studies demonstrated that CD36 is primarily necessary to form chylomicrons [40] and regulate the output of very low-density lipoproteins (VLDL) [41]. The latter function of CD36 might explain why Cd36^-/-^ mice used in the current study had, on average, 51% higher triglyceride levels in their livers and 21% lower serum triglycerides than the corresponding WT mice. These findings fit well to data which showed that CD36 deletion exacerbated the steatosis in ob/ob mice by impairing the hepatic triglyceride secretion via VLDL [42]. Interestingly, we observed that Cd36^-/-^ mice showed significantly lower 25(OH)D_3_-d_3_ levels in the serum than WT mice. It should be noted that vitamin D binding protein, which functions as a vitamin D carrier, was found to be present in VLDL [43]. Thus, we assume that the lower serum and kidney levels of 25(OH)D_3_-d_3_ in the Cd36^-/-^ mice could have been caused by a reduced transport of 25(OH)D from liver via VLDL into the circulation. Surprisingly, we found not only lower levels of 25(OH)D_3_-d_3_ in serum, but also in the livers of Cd36^-/-^ mice which are not indicative of an impaired transfer of this vitamin D metabolite into the circulation. Interestingly, low serum levels of 25(OH)D have also been observed in patients suffering from nonalcoholic liver diseases compared to those of healthy subjects and in mice developing fatty livers in response to a high-fat diet compared to those fed a standard chow diet [44]. Thus, we may speculate that the accumulation of liver lipids in the Cd36^-/-^ mice could have hampered the 25(OH)D synthesis, although the mRNA abundance of the vitamin D hydroxylases in the livers are not indicative of an affected expression of these enzymes. Comparable to the findings in the Srb1^-/-^ mice, the reduced 25(OH)D levels in the Cd36^-/-^ mice were associated with an increased mRNA expression of renal *Cyp27b1* mRNA to prevent a decline in 1,25(OH)_2_D.

Finally, we investigated the impact of ABC-G5/G8 on the levels of vitamin D metabolites in serum and tissues. The ABC-G5/G8 transporters are known to exert two common functions: the reverse intestinal transport of sterols [16] and the hepatic excretion of sterols into bile [17,45]. The significantly higher levels of serum lipids found in the Abcg5/g8^-/-^ mice of the current study correspond to data on polymorphic variants of ABC-G5/G8 in humans that have been associated with elevated levels of circulating cholesterol and triglycerides [46,47]. The present findings show that Abcg5/g8^-/-^ mice had higher serum and liver levels of vitamin D_3_-d_3_ and increased serum and renal levels of 25(OH)D_3_-d_3_ compared to WT mice. The improved vitamin D status of the Abcg5/g8^-/-^ mice support the hypothesis that ABC-G5/G8 can also function as reverse vitamin D transporters. Thus, we speculate that the increased serum and liver levels of vitamin D_3_-d_3_ in Abcg5/g8^-/-^ mice were attributed to a higher absorption rate and reduced excretion of vitamin D_3_-d_3_ into bile. We further assume that the increase in 25(OH)D_3_-d_3_ was caused not only by higher levels of available vitamin D_3_-d_3_ but also by an increase in the expression of hepatic vitamin D hydroxylases because Abcg5/g8^-/-^ mice showed a higher mRNA abundance of *Cyp27a1* in their livers than WT mice. In this context, it should be mentioned that Abcg5/g8^-/-^ mice had higher liver weights than WT mice, despite having unchanged liver lipids. Increased liver weights were also observed in previous studies using Abcg5/g8^-/-^ mice that were fed a diet with 0.2% plant sterols [48] or Abcg5^-/-^ mice that were fed a commercial chow diet [49]. The authors of the first study concluded that plant sterol accumulation in Abcg5/g8^-/-^ mice could have caused toxic liver effects. Whether traces of naturally occurring phytosterols in the dietary components that were used for preparation of the experimental diets had caused the increase in the liver weights of the Abcg5/g8^-/-^ mice in the current study remain uncertain, but data are not indicative of any injury in the livers of Abcg5/g8^-/-^ mice as they did not show increased serum transaminases ASAT and ALAT compared to the corresponding controls.

In contrast to Srb1^-/-^ and Cd36^-/-^ mice, the expression of renal *Cyp27b1* was not affected in the Abcg5/g8^-/-^ mice, indicating an improved vitamin D status in these animals. Based on the current findings that suggest a role of ABC-G5/G8 for reverse intestinal vitamin D transport, it should be kept in mind that any stimulation of ABC-G5/G8 transport may probably impair vitamin D status. Phytosterols are known stimulators of the intestinal expression and activity of ABC-G5/G8 [19]. The assumption that ABC-G5/G8 can deteriorate vitamin D status is corroborated by previous data that showed a reduced vitamin D_3_ bioavailability in mice treated with phytosterols [20].

Finally, we would like to point out some limitations of this study. First, we included only male but not female mice in this study. Although sex-specific differences are, so far, not described for SR-B1 and ABC-G5/G8, data indicate that there are sex-specific differences in the expression of CD36 [50]. Thus, future studies investigating the role of CD36 for vitamin D metabolism should include males and females. A second limitation was the restriction of food intake. Although data show that all mice gained weight during the experimental period, their final body weights were slightly lower than those of age-adjusted observed from age-adjusted WT mice which were fed a standard chow diet *ad libitum*. Vitamin D_3_-d_3_ supplementation via oral gavage might be an option to avoid diet-related impact on lipid metabolism in these mouse models. We would also like to emphasize that the findings only apply to oral not endogenously synthesized vitamin D.

## 5. Conclusions

To conclude, the current data highlight the role of these transporters in the uptake, tissue distribution and activation of vitamin D. The data suggest that SR-B1 is important for the uptake of ingested vitamin D into the liver for the synthesis of 25(OH)D. Mice deficient in CD36 were characterized by moderately reduced 25(OH)D levels, but otherwise unchanged levels of vitamin D metabolites. It is tempting to speculate that Cd36^-/-^ mice showed an impaired hepatic synthesis of 25(OH)D and transport into the circulation. Finally, the sterol exporters ABC-G5/G8 appear to be also involved in reverse vitamin D transport. This would explain the higher vitamin D concentrations in serum and tissues that we found in the Abcg5/g8^-/-^ mice compared to WT mice.

## Figures and Tables

**Table 1:** Food intake, final body weights, circulating lipids, serum transaminases and lipid levels in liver of SR-B1-, CD36- and ABC-G5/G8-deficient mice compared to the corresponding WT mice.

	WT	Srb1^-/-^	Cd36^-/-^	WT vs. Srb1^-/-^ *p* Value	WT vs. Cd36^-/-^ *p* Value	WT	Abcg5/g8^-/-^	WT vs. Abcg5/g8^-/-^ *p* Value
Food intake (g/d)	2.4 ± 0.0	2.2 ± 0.2	2.4 ± 0.0	0.018	0.549	2.4 ± 0.0	2.4 ± 0.0	0.186
Body weight gain (g)	4.8 ± 1.5	3.1 ± 1.1	3.9 ± 0.9	0.073	0.264	3.2 ± 1.1	4.1 ± 1.3	0.287
Final body weight (g) *	26.6 ± 1.6	24.0 ± 1.9	26.8 ± 1.8	0.043	0.869	28.6 ± 2.6	28.3 ± 1.8	0.808
**Serum**								
Triglycerides (mmol/L)	0.92 ± 0.06	1.34 ± 0.25	0.73 ± 0.06	0.005	0.001	0.88 ± 0.30	2.20 ± 0.38	<0.001
Cholesterol (mmol/L)	3.67 ± 0.51	10.73 ± 0.99	4.85 ± 0.46	<0.001	0.004	3.37 ± 1.30	6.02 ± 0.58	0.001
7-DHC (nmol/L)	136 ± 12	973 ± 250	180 ± 25	<0.001	0.005	174 ± 54	236 ± 19	0.041
ASAT (U/L)	222 ± 41	232 ± 49	217 ± 77	0.734	0.900	283 ± 30	226 ± 99	0.222
ALAT (U/L)	28.7 ± 6.8	40.5 ± 14.1	35.6 ± 9.7	0.120	0.222	37.8 ± 14.6	46.0 ± 6.3	0.516
**Liver**								
Absolute weight (g)	0.91 ± 0.05	0.83 ± 0.08	1.04 ± 0.13	0.078	0.051	0.87 ± 0.18	1.23 ± 0.11	0.003
Relative weight (g/100 g body weight)	4.06 ± 0.41	3.85 ± 0.23	4.40 ± 0.39	0.339	0.210	3.16 ± 0.49	4.83 ± 0.46	<0.001
Triglycerides (mg/g)	60.8 ± 12.1	65.2 ± 44.9	91.9 ± 36.7	0.837	0.102	140.4 ± 60.9	128.9 ± 41.1	0.730
Cholesterol (mg/g)	3.79 ± 0.54	3.63 ± 0.83	2.89 ± 0.47	0.737	0.027	4.76 ± 1.27	3.38 ± 0.85	0.067

* Body weight range of age-adjusted WT mice on standard chow: 26.9–33.0 g (data provided by the Jackson Laboratory, https://www.jax.org/strain/000664). Data are presented as the means ± SD, *n* = 5–7. All mice were fed diets with 25 µg/kg triple-deuterated vitamin D_3_ (vitamin D_3_-d_3_) for six weeks. Each group of knockout mice was compared by their corresponding wild-type (WT) mice by Student’s *t* test. 7-DHC, 7-dehydrocholesterol; Abcg5/g8^-/-^, ATP-binding cassette transporters G5/G8 knockout mice; ALAT, alanine transaminase; ASAT, aspartate transaminase; Cd36^-/-^, cluster determinant 36 knockout mice; Srb1^-/-^, scavenger receptor class B type 1 knockout mice.

## Abbreviations

1,25(OH)_2_D, 1α,25-dihydroxyvitamin D; 25(OH)D, 25-hydroxyvitamin D; 25(OH)D_3_-d_3_, triple-deuterated 25-hydroxyvitamin D_3_; 7-DHC, 7-dehydrocholesterol; ABC-G5/G8, ATP-binding cassette transporters G5/G8; ALAT, alanine aminotransferase; ASAT, aspartate aminotransferase; CD36, cluster determinant 36; Cyp27a1, sterol 27-hydroxylase; Cyp27b1, 25-hydroxyvitamin D 1α-hydroxylase; Cyp2r1, vitamin D 25-hydroxylase; Gapdh, glyceraldehyde-3-phosphate dehydrogenase; HDL, high-density lipoprotein; Hprt, hypoxanthine phosphoribosyltransferase; NPC1L1, Niemann-Pick C1-like protein 1; Rplp0, ribosomal protein lateral stalk subunit P0; SR-B1, scavenger receptor class B type 1; vitamin D_3_-d_3_, triple-deuterated vitamin D_3_; VLDL, very low-density lipoproteins; WT, wild-type.
